# Whole-genome sequencing of two probands with hereditary spastic paraplegia reveals novel splice-donor region variant and known pathogenic variant in *SPG11*

**DOI:** 10.1101/mcs.a001248

**Published:** 2016-11

**Authors:** Allen Chi-Shing Yu, Anne Yin-Yan Chan, Wing Chi Au, Yun Shen, Ting Fung Chan, Ho-Yin Edwin Chan

**Affiliations:** 1 School of Life Sciences, Faculty of Science, The Chinese University of Hong Kong, Shatin, N.T., Hong Kong SAR;; 2 Partner State Key Laboratory of Agrobiotechnology, The Chinese University of Hong Kong, Shatin, N.T., Hong Kong SAR;; 3 Division of Neurology, Department of Medicine and Therapeutics, Faculty of Medicine, The Chinese University of Hong Kong, Shatin, N.T., Hong Kong SAR;; 4 Gerald Choa Neuroscience Centre, The Chinese University of Hong Kong, Shatin, N.T., Hong Kong SAR

**Keywords:** gait imbalance, slowed slurred speech, spastic dysarthria, spastic gait, spastic paraparesis, spastic paraplegia

## Abstract

Hereditary spastic paraplegias (HSPs) are a group of heterogeneous neurodegenerative disorders, which are often presented with overlapping phenotypes such as progressive paraparesis and spasticity. To assist the diagnosis of HSP subtypes, next-generation sequencing is often used to provide supporting evidence. In this study, we report the case of two probands from the same family with HSP symptoms, including bilateral lower limb weakness, unsteady gait, cognitive decline, dysarthria, and slurring of speech since the age of 14. Subsequent whole-genome sequencing revealed that the patients are compound heterozygous for variants in the *SPG11* gene, including the paternally inherited c.6856C>T (p.Arg2286*) variant and the novel maternally inherited c.2316+5G>A splice-donor region variant. Variants in *SPG11* are the common cause of autosomal recessive spastic paraplegia type 11. According to the ClinVar database, there are already 101 reported pathogenic variants in *SPG11* that are associated with HSPs. To our knowledge, this is the first report of *SPG11* variants in our local population. The novel splice variant identified in this study enriches the catalog of *SPG11* variants, potentially leading to better genetic diagnosis of HSPs.

## CASE PRESENTATION

Spastic paraplegia type 11 (SPG11) is a type of autosomal recessive neurological disease characterized by the progression of lower limb muscle stiffness and spasticity and often accompanied with thinning of the corpus callosum, intellectual disability, neuropathy, and a variety of neurological symptoms. Onset time is childhood to early adult (Shibasaki et al. 2000; Casali et al. 2004; Winner et al. 2004; Olmez et al. 2006; Hehr et al. 2007). A majority of SPG11 cases are caused by homozygous recessive or compound heterozygous variants in the *SPG11* gene (Stevanin et al. 2007, 2008), which codes for the Spatacsin protein with a role in axonal maintenance, cargo trafficking (Pérez-Brangulí et al. 2014), and autophagy (Chang et al. 2014). Because of overlapping phenotypes in different hereditary spastic paraplegia (HSP) subtypes (Pensato et al. 2014), diagnosis of SPG11 is often supplemented with evidence from molecular genetics testing. In particular, next-generation sequencing is gaining traction as a tool for assisting the diagnosis and treatment of neurological diseases (Tsoi et al. 2014; Petrovski et al. 2015; Yang et al. 2015; Ye et al. 2015).

**Figure 1.**
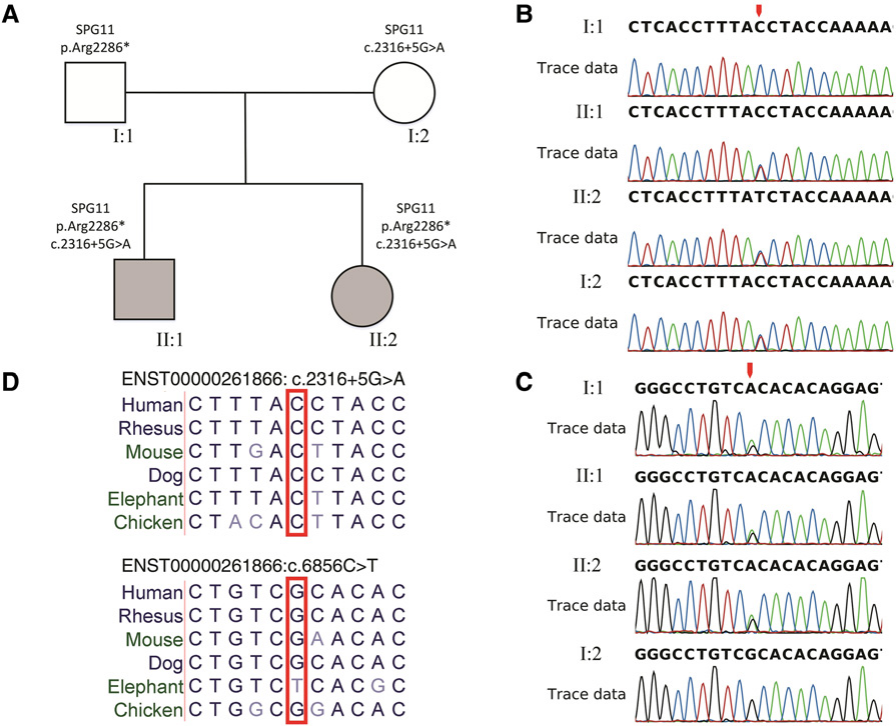
Compound heterozygous variants in *SPG11* in probands. (*A*) Pedigree of family under study. The two probands inherited compound heterozygous variants (ENST00000261866:c.6856C>T;p.Arg2286* and ENST00000261866:c.2316+5G>A) from unaffected parents. Sanger sequencing validation of (*B*) the ENST00000261866:c.2316+5G>A variant and (*C*) the ENST00000261866:c.6856C>T;p.Arg2286* variant. Mutated positions are marked with red arrows. (*D*) Multiz alignments of vertebrates showed a high degree of conservation at the mutated location.

We report the case study of a family with two probands that showed HSP symptoms (Fig. 1A). Both parents were asymptomatic without history of consanguineous marriages. The age of disease onset for the probands was 14, when both of them showed bilateral lower limb weakness and unsteady gait (Table 1). Subsequently, they developed slurring of speech, dysarthria, and cognitive decline. The elder female proband (II:2) presented with slower disease progression, and she is still able to walk with aids. On the other hand, the younger male proband (II:1) suffered from rapid deterioration over a few years after onset and became bedridden. Extensive workup had been done including serum copper, cortisol, cholestanol, campesterol, stigmasterol, and β-sitosterol levels; however, all of these indicators were normal. Further skin biopsy and urine testing for porphyrin also showed negative results. The magnetic resonance imaging (MRI) brain imaging for II:1 showed mild cerebral and cerebellar atrophy at the age of 25, which is compatible with his symptoms of cognitive decline and unsteady gait (Supplemental Fig. 1).

**Table 1.** Clinical parameters of the two probands investigated in this study

	II:2	II:1
Age	31	29
Gender	Female	Male
Age of onset	14 years old	14 years old
Presenting symptoms	Unsteady gait	Unsteady gait
Cognitive decline	Yes	Yes
Psychosis	No	Yes
Spasticity	Yes	Yes

## METHODS

To elucidate the diagnosis of the HSP subtype and potentially inform clinical decisions, whole-genome sequencing was performed using the Illumina HiSeq X Ten platform for all four family members, including the two probands and two unaffected parents. On average, ∼214.8 Gb (∼66.4× depth of coverage) of DNA sequence was generated for each individual (Supplemental Table 1). The short read sequences were aligned to the human genome (version GRCh38) using Burrows–Wheeler alignment (BWA) (version 0.7.12) (Li and Durbin 2009), followed by polymerase chain reaction (PCR) duplicate marking, local realignment around indels, and base quality score recalibration using Picard (version 1.141) tools and the Genome Analysis Toolkit (GATK, version 3.4) (McKenna et al. 2010). Sequence variants and small indels were called according to the best practices for using the GATK HaplotypeCaller (McKenna et al. 2010), resulting in ∼4,790,000 sequence variants and small indels. Because HSP is a rare disease affecting 1–10 in 100,000 individuals depending on the geographical location (Fink 2006), common variants with a minor allele frequency (MAF) >5% were filtered, leaving ∼450,000 variants on average per individual (Supplemental Table 1). Finally, variant annotation was performed using SnpEff (Cingolani et al. 2012) and dbNSFP (Database for Nonsynonymous SNPs’ Functional Predictions; Liu et al. 2013). Published guidelines from the American College of Medical Genetics and Genomics (ACMG) were used for interpretation of the variants (Richards et al. 2015). Copy-number variants and structural variants were called using LUMPY (version 0.2.11) (Layer et al. 2014), yet none of these larger scale variants match the inheritance pattern. Sequence variants focused in this study were validated using conventional Sanger sequencing analysis (Table 2).

**Table 2.** List of variants in *SPG11*

Genomic location (GRCh38)	dbSNP/ClinVar	HGVS	1000G MAF	Variant interpretation	CADD	MutationTaster	I:2	I:1	II:1	II:2
Chr15: 44565997	rs312262785/41353	ENST00000261866:c.6856C>T; p.Arg2286*	0.0002	Pathogenic (PVS1, PM1, PM2, PP3, PP4)	Pathogenic (47)	Disease causing (1)	N/A	0/1	0/1	0/1
Chr15: 44622723	rs879255274/252959	ENST00000261866:c.2316+5G>A ENST00000559193:c.2321G>A; p.Gly774Asp	N/A	Likely pathogenic (PM2, PM3, PP1, PP3, PP4)	Pathogenic (19.36)	Disease causing (1)	0/1	N/A	0/1	0/1
Chr15: 44651599	rs3759873/130364	ENST00000261866:c.1348A>G; p.Ile450Val	0.0389	Benign (BA1, BS1, BS4, BP4)	Neutral (0.002)	Polymorphism (0.994)	N/A	0/1	N/A	N/A

Genotypes for each family member are shown in the right-most columns, in which 0/1 represents heterozygous. Parenthetical codes in the Variant interpretation column denote the pathogenic criteria in the ACMG (American College of Genetics and Genomics) guidelines 2015 (Richards et al. 2015). Predicted functional impact on the transcript and protein was calculated by SnpEff, CADD, and MutationTaster. Numbers in the CADD column denote the degree of pathogenicity in Phred scale. Numbers in the MutationTaster column denote the confidence of pathogenicity classification, in which 1 is the most confident and 0 is the least confident.

dbSNP, Database for Short Genetic Variations; HGVS, Human Genome Variation Society; 1000G, 1000 Genomes; MAF minor allele frequency; CADD, Combined Annotation-Dependent Depletion; N/A, not applicable.

## VARIANT INTERPRETATION

After annotation of variants in accordance to the ACMG standards (Richards et al. 2015), one pathogenic nonsense variant was discovered in *SPG11* (ENST00000261866:c.6856C>T; p.Arg2286*). The variant is extremely rare, where the global MAF of the variant is 0.0002 (1/5008) in the 1000 Genomes Project Phase III or 1.664 × 10^−5^ (2/120202) in the Exome Aggregation Consortium (ExAC). The p.Arg2286* variant is known to be associated with autosomal recessive SPG11 (Denora et al. 2009) and recorded as a pathogenic variant in the ClinVar database (Variation ID: 41353). Subsequent Sanger sequencing validation confirmed that all probands are heterozygous carriers of the allele (Fig. 1A,B). However, the heterozygous variant alone cannot fully explain the observed autosomal recessive inheritance pattern, because the unaffected father also carries the p.Arg2286* variant (Fig. 1A,B). This suggests the possibility of compound heterozygosity, in which more than one variant contributes to the autosomal recessive pattern.

To explore the possibility of compound heterozygosity, we expanded the search of *SPG11* sequence variants and indels to the ACMG “Likely Pathogenic” and “Unknown Significance” tiers. One heterozygous variant in the splice-donor region of *SPG11* exon 12 (ENST00000261866:c.2316+5G>A) was found to be shared by two probands and the unaffected mother, which was subsequently validated using Sanger sequencing (Fig. 1C). This novel variant was not described in the 1000 Genomes Project Phase III, the National Heart, Lung, and Blood Institute (NHLBI) Exome Sequencing Project (ESP), nor the ExAC database. Based on multiple sequence alignment of *SPG11* sequences from six vertebrates, c.6856C>T and c.2316+5G>A variants were located in a conserved location (Fig. 1D). The variant's impact on splice pattern was further assessed using Human Splicing Finder (HSF) (Desmet et al. 2009) and Alternative Splice Site Predictor (ASSP) (Wang and Marín 2006). HSF showed that the mutant splice site would be 92% weaker than wild type based on the MaxEnt model, whereas ASSP suggested that the variant would lead to the loss of the splice-donor site. Because of alternative splicing, this novel variant can also be found in the coding region of the alternative transcript (ENST00000559193:c.2321G>A; p.Gly774Asp). However, ENST00000559193 is not expressed in brain, whereas ENST00000261866 is the most abundant transcript among those with Transcription Support Level 1 in the Genotype-Tissue Expression (GTEx) project (Lonsdale et al. 2013). This suggests that the variant mainly exerts its impact through ENST00000261866:c.2316+5G>A. The novel maternally inherited c.2316+5G>A variant, in combination with the paternally inherited c.6856C>T (p.Arg2286*) variant, supports the compound heterozygous diagnosis of SPG11.

## SUMMARY

The *SPG11* gene encodes for the Spatacsin protein, yet the detailed molecular function of Spatacsin is not well understood. It was suggested that axonal defects were observed in patients with nonsense and splice variants in *SPG11*, which is further supported by a functional study using a mouse model (Pérez-Brangulí et al. 2014). Loss of Spatacsin also causes accumulation of autolysosomes and deprivation of free lysosomes, thereby disrupting the autophagic lysosome reformation pathway, ultimately leading to neurodegeneration (Chang et al. 2014). In this study, we have identified compound heterozygous variants in *SPG11* that were predicted to cause truncation of the corresponding protein. The p.Arg2286* nonsense variant was previously reported to be associated with SPG11 (Denora et al. 2009); while in the vicinity of the novel c.2316+5G>A variant identified in this study, a splice variant (c.2316+1G>A) was previously linked to SPG11 (Stevanin et al. 2008). In a recent large-scale investigation of SPG11 cases in London, 79.5% of *SPG11* variants were found to be nonsense, frameshift, or splice-site variants that could cause large-scale amino acid sequence changes (Kara et al. 2016). Because both variants identified in this study were predicted to cause the truncation of Spatacsin, findings in this study further supported the major role of loss-of-function variants in SPG11 pathogenesis (Pensato et al. 2014; Pérez-Brangulí et al. 2014). To our knowledge, our study is the first report of *SPG11* variants in the Hong Kong population. The novel splice variant identified in this study expands the repertoire of *SPG11* variants, facilitating the molecular genetic testing of HSP.

## ADDITIONAL INFORMATION

### Data Deposition and Access

Raw sequencing data were deposited to the European Genome-phenome Archive (EGA; http://www.ebi.ac.uk/ega) under accession number EGAS00001001849. The variant was deposited in ClinVar (http://www.ncbi.nlm.nih.gov/clinvar/) under accession number SCV000292372.

### Ethics Statement

Informed and signed consent forms were obtained for all sequenced individuals of this study. The project is approved by The Joint Chinese University of Hong Kong–New Territories East Cluster Clinical Research Ethics Committee (CRE-2012.361).

### Author Contributions

Patients were recruited and phenotyped by A.Y.-Y.C. and W.C.A. Data analysis, interpretation, and validation were performed by A.C.-S.Y. and Y.S. The manuscript was prepared by A.C.-S.Y., A.Y.-Y.C., Y.S., H.-Y.E.C., and T.F.C. All authors contributed to the reviewing of the final version.

### Funding

This work was partially supported by the Chow Tai Fook Charity Foundation (6903898), Hong Kong Spinocerebellar Ataxia Association (6903291), and Gerald Choa Neuroscience Centre (7105306) grants to H.-Y.E.C. A.C.-S.Y. and T.F.C. are supported by the Research Grants Council (RGC) General Research Fund (GRF14102014) and Collaborative Research Fund (CRF: C4042-14G).

### Competing Interest Statement

T.F.C. and H.-Y.E.C. are scientific advisors of Codex Genetics Limited (Codex), and A.C.-S.Y. is the genetic scientist at the same company. However, neither financial relationships nor activities with Codex appear to have influenced the submitted work.

## Supplementary Material

Supplemental Material
