# Supplementary material for: Whole-genome sequencing of two probands with hereditary spastic paraplegia reveals novel splice-donor region variant and known pathogenic variant in SPG11
Source: Cold Spring Harb Mol Case Stud. 2016 Nov;2(6):a001248. doi: 10.1101/mcs.a001248 (PMC5111012; doi:10.1101/mcs.a001248)
Supplement: Supplemental Material [file supp_2_6_a001248__index.html]

Whole-genome sequencing of two probands with hereditary spastic paraplegia reveals novel splice-donor region variant and known pathogenic variant in SPG11 — Supplemental Material 

# Whole-genome sequencing of two probands with hereditary spastic paraplegia reveals novel splice-donor region variant and known pathogenic variant in *SPG11*

## Supplemental Material

**Files in this Data Supplement:**

- Supplemental Figure 1.pdf
- Supplemental Table 1.pdf
