# Supplementary material for: Whole-genome sequencing of two probands with hereditary spastic paraplegia reveals novel splice-donor region variant and known pathogenic variant in SPG11
Source: Cold Spring Harb Mol Case Stud. 2016 Nov;2(6):a001248. doi: 10.1101/mcs.a001248 (PMC5111012; doi:10.1101/mcs.a001248)
Supplement: Supplemental Material [file supp_mcs.a001248_SuppFig1.pdf]

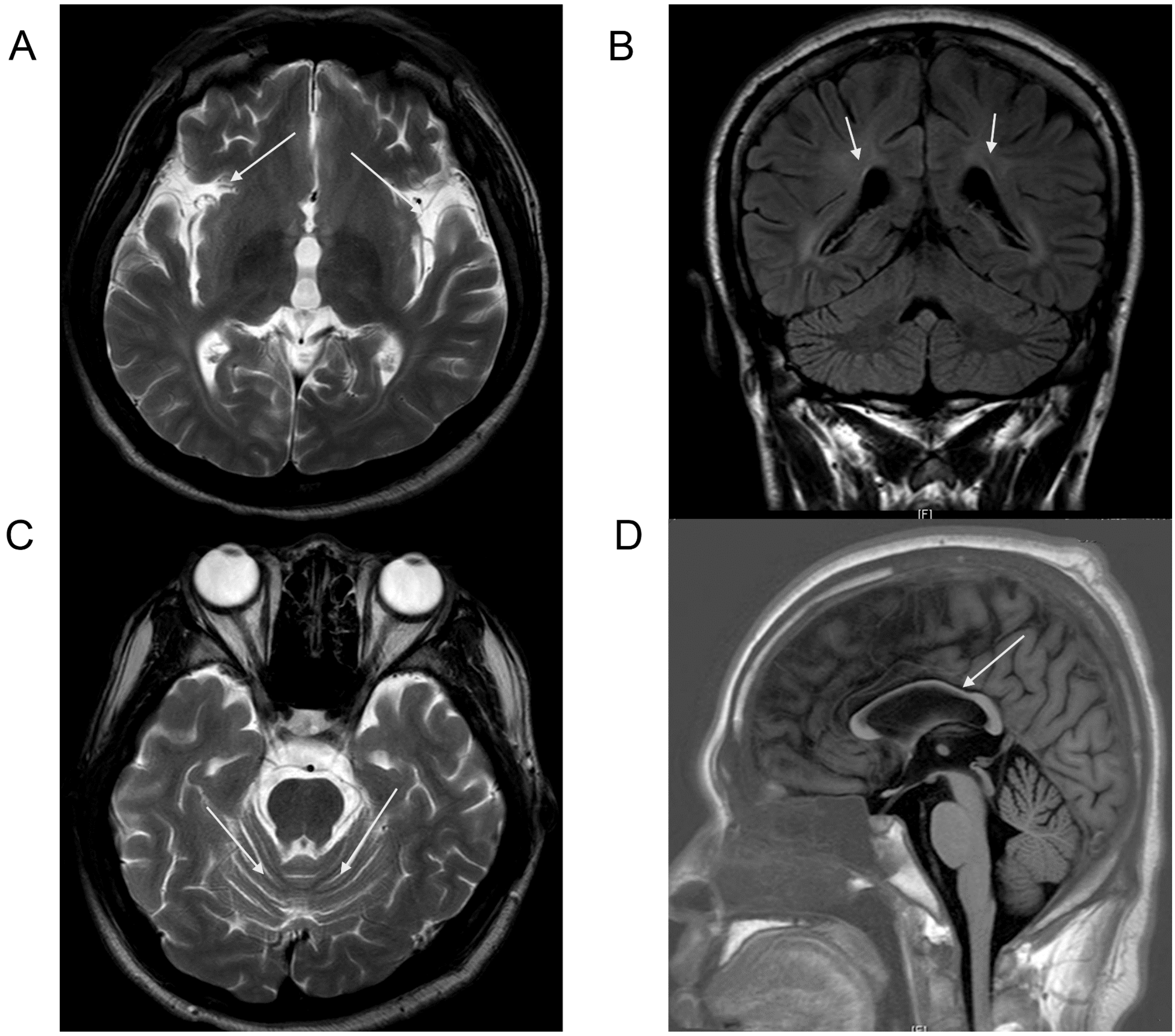

Supplementary figure 1. MRI image of brain showed both cerebral and cerebellar atrophy of proband (II:1). White arrows indicate the sites of abnormalities. (A) Axial T2-Weighted MRI showing bilateral temporal atrophy. (B) Coronal FLAIR image showing periventricular white matter lesions. (C) Axial T2-Weighted MRI showing bilateral cerebellar atrophy. (D) Mid-saggital T1-Weighted MRI image showing a thin corpus callosum with frontal cortical atrophy.
