# Supplementary material for: Whole-genome sequencing of two probands with hereditary spastic paraplegia reveals novel splice-donor region variant and known pathogenic variant in SPG11
Source: Cold Spring Harb Mol Case Stud. 2016 Nov;2(6):a001248. doi: 10.1101/mcs.a001248 (PMC5111012; doi:10.1101/mcs.a001248)
Supplement: Supplemental Material [file supp_mcs.a001248_SuppTable1.pdf]

Sample	I:1	I:2	II:1	II:2
#Total Reads (Million)	1387.95	1561.69	1368.81	1397.71
Aligned Reads (Million)	1279.25	1447.27	1256.06	1295.88
% Aligned Reads	92.17	92.67	91.76	92.71
Aligned Bases (GB)	190.3	214.72	186.41	192.72
Depth of Coverage	61.47	69.36	60.22	62.25
Duplicate reads (Million)	95.129136	101.06036	79.703854	91.452988
% Duplicates	6.59%	6.23%	5.60%	6.30%
SNPs	3925696	3955323	3910033	3943404
INDELs	849617	860070	862920	860168
Ti/Tv	1.97	1.98	1.98	1.98
SNPs (MAF<0.5)	327328	325737	320062	320165
INDELs (MAF<0.5)	126589	129808	132240	129362

Supplementary Table 1. Whole-genome sequencing and variant calling statistics
